# Effect of Food Intake on Vortex Formation Time as a Measurement of Diastolic Left Ventricular Function

**DOI:** 10.3390/jcm14165783

**Published:** 2025-08-15

**Authors:** Sarah Smith, Andreas Malmgren, Ylva Gårdinger, Joanna Hlebowicz, Magnus Dencker

**Affiliations:** 1 Department of Medicine, Trelleborg Hospital, 231 85 Trelleborg, Sweden; 2 Department of Medical Imaging and Physiology, Skåne University Hospital, Lund University, 205 02 Malmo, Sweden; andreas.malmgren@skane.se (A.M.);; 3 Department of Cardiology, Skåne University Hospital, Lund University, 222 42 Lund, Sweden

**Keywords:** food intake, left ventricular diastolic function, transthoracic echocardiography, vortex formation time

## Abstract

**Objectives:** The aim of the present study was to assess if vortex formation time (VFT) as a measurement of left ventricular (LV) diastolic function is affected by food intake and related to age and sex. **Methods:** Healthy participants were divided into two age groups: younger (median age: 25 years) and older (median age: 68 years). Transthoracic echocardiography (TTE) examinations were performed during fasting as well as 30 min after a standardized meal. Measurements of the TTE images were performed off-line for the calculation of VFT. **Results:** There were no differences in VFT between men and women regardless of age. There was a significant increase in VFT from a median value of 2.0 (1.5–2.5) to a median value of 2.3 (1.5–2.0) after food intake in the older study group (*p* < 0.001). This was not observed in the younger study group, which had a median value of VFT of 2.5 (2.1–3.0) before food intake and a median value of VFT of 2.5 (2.2–3.1) after food intake (*p* = 0.369). Furthermore, VFT was significantly higher in the younger study group, i.e., 2.5 (2.1–3.0), compared to the older study group, i.e., 2.0 (1.5–2.5), before food intake (*p* = 0.011), but not after food intake, with a median value of VFT in the younger group of 2.5 (2.2–3.1) and the older group of 2.3 (1.5–2.9) (*p* = 0.172). **Conclusions:** Our findings suggest that VFT is affected by age, not by sex. Moreover, VFT is affected by food intake only in elderly subjects.

## 1. Introduction

Food intake is known to result in significant hemodynamic changes that affect the cardiovascular system in different ways [1,2,3,4,5,6,7]. Previous studies have shown that different functional parameters of the left ventricle (LV) such as cardiac output (CO), stroke volume (SV), and diastolic function investigated with transthoracic echocardiography (TTE) are affected by food intake [1,2,3,4,5,6,7]. A meal can influence cardiovascular function in a manner similar to an arterial vasodilator drug. It results in a decrease in systemic vascular resistance and a secondary increase in CO [1,8]. Food intake and preload vary throughout the day, which may affect the results of the different hemodynamic parameters evaluated with TTE. Currently, there are no TTE examination guidelines regarding food intake [6].

TTE is a cornerstone used in daily clinical practice for the evaluation of LV systolic and diastolic function and the hemodynamic assessments of the heart [3,4,9,10]. Assessing diastolic function using TTE involves considering various parameters, including the patient’s age [11]. Studies suggest that the current TTE measurements of LV diastolic function have limitations [12,13], making evaluations challenging. During the rapid filling phase of diastole, blood moves like a rotating vortex ring in the LV, which finally moves towards the left ventricle outflow tract (LVOT) [14,15]. The optimal formation of the vortex ring depends on LV relaxation that generates adequate suction, a normal mitral valve that allows blood volume to pass to the LV, and a normal conduction system in the myocardium that enables harmonious cardiac contraction. It is, therefore, expected that changes in the structure and function of the LV affect the formation of the vortex ring and thus vortex formation time (VFT), which is a dimensionless index used to quantify the process of vortex formation that occurs in the early diastole. This index may lead to a better understanding of diastolic LV function and the filling efficiency of the LV [14,15,16]. The form of the vortex ring may also be used to differentiate between different degrees of diastolic dysfunction and thus can provide useful information about the prognosis in patients with diastolic dysfunction [17]. Age is known to significantly impact the cardiovascular system, influencing both its physiological functions and pathological conditions [3]. With increasing age, relaxation is prolonged and myocardial stiffness increases [18], leading to an elevated ventricular end-diastolic pressure (LVEDP) at rest and during exercise [19,20]. In the elderly, a common characteristic is a decrease in early diastolic filling. To maintain CO, there is often a compensatory increase in late diastolic filling through increased atrial contraction [19].

Both food intake [1,2,3,4,5,6,7] and age [3] affect the cardiovascular system in different ways. Vortex formation time (VFT) has been suggested as a potential indicator for cardiac health and has been compared across different patient groups [15]. The aim of the present study was to assess whether VFT as a measurement of diastolic LV function is affected by food intake and related to age and sex.

## 2. Material and Method

### 2.1. Study Population

This study was based on already collected material from previous studies [3,4]. Two healthy populations were included in this study: younger (12 female and 11 male) and older (15 female and 15 male) subjects. The younger group had healthy volunteers over the age of 18, and the older group had randomly selected and recruited volunteers from the Swedish population register who were between the ages of 65 and 70 years. None of the participants were taking any cardiovascular medication or had symptoms or a history of cardiovascular diseases. Other exclusion criteria were inappropriate echocardiographic acoustic windows and non-sinus rhythm.

### 2.2. Procedure

Examinations were performed in the morning after overnight fasting. Height and weight were measured. Body surface area (BSA) [21] and body mass index (BMI) were calculated. In the younger population, blood pressure was measured using a conventional (mechanical) sphygmomanometer with an aneroid manometer and a stethoscope. Phase I and V Korotkoff sounds were used to identify systolic and diastolic blood pressure (SBP and DBP), respectively. In the older population, blood pressure was measured with an Omron M8 Comfort device (Omron healthcare, Kyoto, Japan). Blood pressure and resting heart rate (HR) were measured in the supine position after 15 min of rest. A baseline TTE was performed, and afterwards, the participants ate a standardized meal consisting of 300 g of rice pudding. In the younger population (AXA, Goda Gröten, Risgrynsgröt; Lantmännen, AXA, Järna, Sweden) and in the older population (Felix Risgröt, Orkla Food, Malmö, Sweden), the caloric value of the meal was 330 kcal, of which 58–60% came from carbohydrates (both brands of pudding contained 48 g), 10–13% from protein (AXA 9 g and Felix 11 g), and 27–32% from fat (AXA 12 g and Felix and 10 g). The switch from AXA, Goda Gröten, to Felix Risgröt was made because the prior was discontinued in between the studies. They then returned to the supine position, and TTE, blood pressure, and HR were measured 30 min after the meal. The selection of 30 min was based on previous hemodynamic studies on the effect of food intake [7].

### 2.3. Vortex Formation and Vortex Formation Time

Echocardiographic particle image velocimetry (PIV) is a modality that can visualize and quantify LV flow patterns in vivo. Studies performed with this modality have shown that, during early diastole, a larger vortex ring forms behind the anterior mitral valve and fills almost the entire LV. In an apical four-chamber echocardiographic view, the vortex ring has a counterclockwise rotation, in which the vector of the vortex ring is directed towards the posterolateral wall. During atrial contraction, the blood flow from the left atrium (LA) is directed more laterally, forming a secondary smaller vortex ring with the same rotation. During early systole, blood flow is directed towards the outflow tract and is unidirectional and laminar [22].

Vortex formation time has been characterized in vitro using a piston/cylinder arrangement in a water tank. Based on the mean velocity of the flow (U_t_), the liquid duration (t), and the diameter (D) of the orifice through which the flow passes, an index of vortex formation has been defined according to the following equation [15,23]:

$$T=\frac{U_{t\;}\times \;t}{D}=\frac{L}{D}$$

The equation can thus be simplified as a ratio of the length (L) to the diameter of the flow (D) [15]. When VFT reaches T ≈ 4, the vortex ring reaches its maximum and does not continue to grow, according to previous studies [15,23,24,25]. By considering some factors that affect the inflow of blood from the LA to the LV, the equation can be modified to also constitute a dimensionless measure for the vortex ring formed in the LV. Since the flow through the mitral valve is related to LV function, an index has been designed based on the left ventricular ejection fraction (LVEF) according to the following equation [15]:

$$VFT=\frac{4\times (1-\beta )}{\pi }\times \alpha^{3}\times LVEF$$

The LVEF is expressed as a percentage and is a measure of the LV systolic function. Simpson’s biplane method was used [26]. α is the ratio of the end diastolic volume (EDV)^1/3^ to the mitral annulus diameter (MAD), and β is a variable that constitutes the ratio of the volume from the atrial contraction to the total inflow through the mitral valve [15].

$$\alpha =\frac{E{DV}^{\frac{1}{3}}}{MAD}\;\beta =\frac{Velocity\;time\;integral,atrial\;contraction\;({VTI}_{A})}{Velocity\;time\;integral,\;early\;diastole({VTI}_{E})+{VTI}_{A}}$$

### 2.4. Transthoracic Echocardiography (TTE)

A screening TTE examination was performed to rule out any obvious cardiac dysfunction. A TTE was performed with the participants in a left lateral position while a three-lead ECG was recorded continuously. The younger population was examined with Sonos 5500 (Philips, Andover, MA, USA) and the older population with a Philips iE33, S5-1 transducer (Koninklijke Philips N.V. Philips Medical System, Amsterdam, The Netherlands). Each study was examined by one experienced operator. Both an apical four-chamber view and an apical two-chamber view were collected. In the apical four-chamber view, a spectral curve with pulsed-waved Doppler in the mitral inflow as well as a spectral curve with pulsed-waved tissue Doppler in the basal part of the septal and the lateral wall of the LV were obtained.

**Figure 1:**
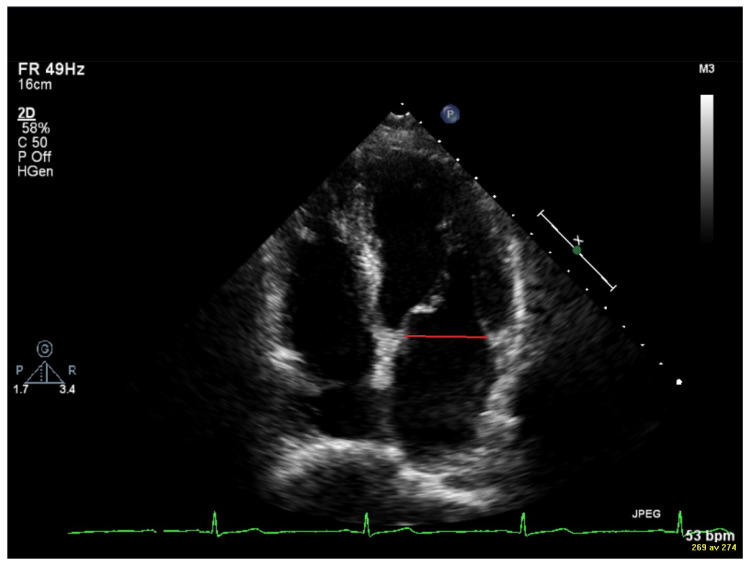
Example of measurement of mitral annulus diameter in early diastole.

**Figure 2:**
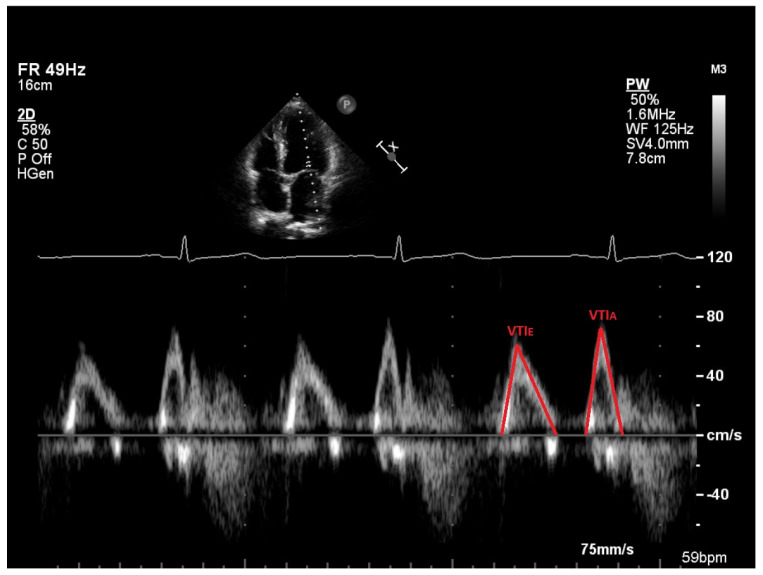
Measurements of velocity time integral (VTI) in early diastole (E-wave) and during atrial contraction (A-wave).

Measurements of the TTE images were made off-line using the software Philips IntelliSpace Cardiovascular 5.2 (Koninklijke Philips N.V. Philips Medical Systems, Eindhoven, The Netherlands). The measurements included the EDV, ESV, mitral annulus diameter (MAD), velocity time integral (VTI) of the E-wave and the A-wave, maximum velocity of the E-wave and A-wave, maximum velocity of the e’-wave, and maximal end-systolic volume of the LA. All measurements were performed on three different heartbeats, and a mean value was calculated and used. The LVEF was calculated with Simpson’s biplane method by manually aligning the endocardium at EDV and ESV in both apical four-chamber and apical two-chamber views. The MAD was measured in early diastole when the mitral valve was open. In the apical four-chamber view, a line was manually drawn between the base of the anterior and posterior mitral leaflets. Figure 1 is an example of measurement of mitral annulus diameter in early diastole. EDV together with MAD are the parameters needed to calculate α. A spectral curve was obtained by pulsed-wave Doppler in the mitral inflow with the sample volume placed between the mitral valve’s tips in an apical four-chamber projection. The maximum velocities of E and A were measured to calculate the E/A ratio. Both the E-wave and the A-wave could also be traced in this spectral curve to obtain the velocity time integral (VTI_E_ and VTI_A_) and the parameters to calculate β. Figure 2 shows measurements of velocity time integral (VTI) in early diastole (E-wave) and during atrial contraction (A-wave). The spectral curve obtained by pulsed-wave tissue Doppler in the basal part of the LV myocardium in both the septal and the lateral wall gives the velocities of é septal and é lateral, and a mean value of é was calculated as the E/é ratio. VFT can be calculated with the parameters LVEF, α, and β. LA volume was measured in end-systole when atria are the largest. To obtain a biplane volume, end-systolic measurements were made in both four-chamber and two-chamber views.

### 2.5. Statistical Analysis

Statistical analysis was performed using SPSS (IBM SPSS Statistics 28, IBM, Armonk, NY, USA) and Wilcoxon matched-pairs test. Regarding the two different populations of age and sex, VFT was compared with a Mann–Whitney U test. Data are presented as median and 25th and 75th quartiles for different variables. A value of *p* < 0.05 was considered statistically significant.

## 3. Results

Table 1 shows descriptive statistics, with median and interquartile ranges (IQR) for both populations. Table 2 shows statistics for the younger population (*n* = 23), and Table 3 shows statistics for the older population (*n* = 30). Table 4 shows the comparison between the older and the younger population regarding VFT and the other parameters involved in the equation used when calculating VFT.

**Figure 3:**
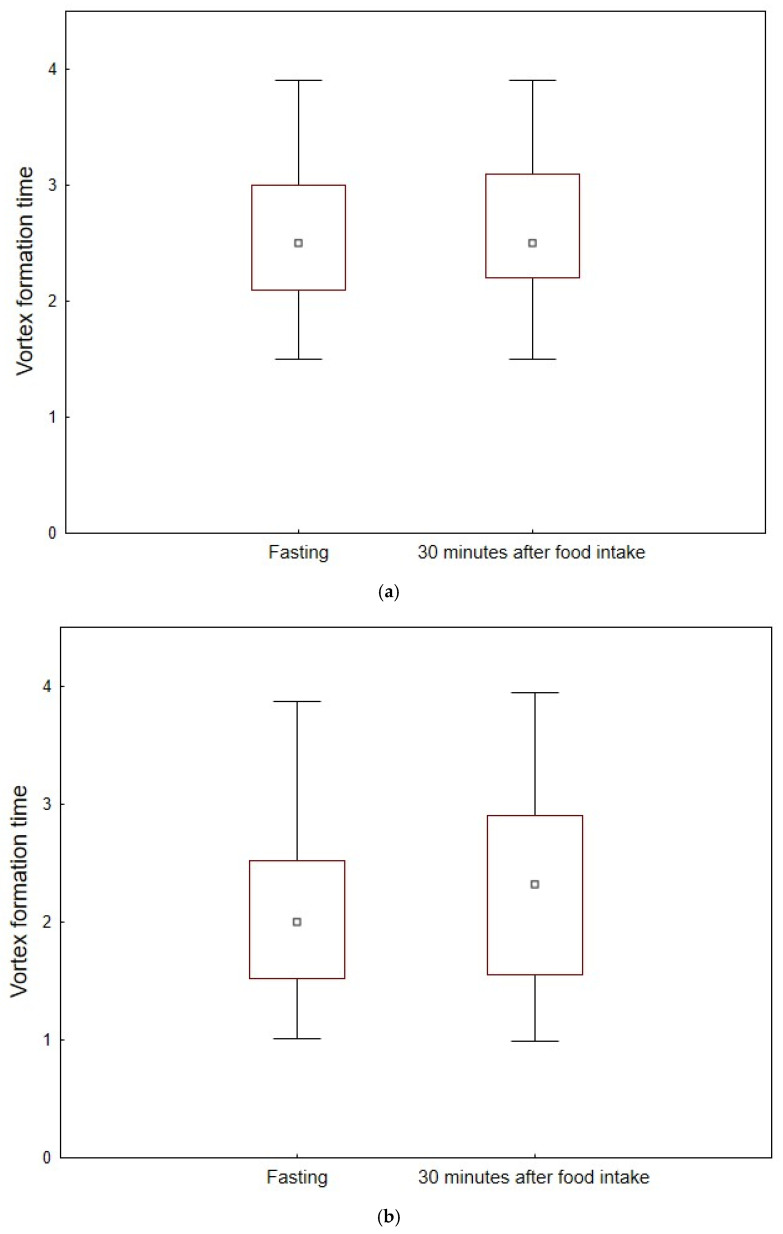
Boxplot for vortex formation time in the younger population (**a**) and in the older population (**b**). Values are mean ± 0.95 confidence intervals of values for fasting and 30 min after food intake.

There were no differences in VFT between men and women regardless of age (*p* > 0.05 for all. There was a significant increase in VFT from a median value of 2.0 (1.5–2.5) to a median value of 2.3 (1.5–2.0) after food intake in the elderly group (*p* < 0.001). This was not observed in the younger group, which had a median value of VFT of 2.5 (2.1–3.0) before food intake and a median value of VFT of 2.5 (2.2–3.1) after food intake (*p* = 0.369). Furthermore, VFT was significantly higher in the younger group, i.e., 2.5 (2.1–3.0), compared to the older group, i.e., 2.0 (1.5–2.5), before food intake (*p* = 0.011). This was not the case after food intake, with a median value of VFT in the younger group of 2.5 (2.2–3.1) and in the older group of 2.3 (1.5–2.9) (*p* = 0.172). Figure 3a,b represent boxplots for vortex formation time in the younger population (a) and in the older population (b). Values are mean ± 0.95 confidence intervals of values for fasting and 30 min after food intake.

Parameters in the younger group that showed a significant difference (*p* < 0.05) after food intake were DBP, HR, LA volume, VTI A, and LVEF (Table 2). Parameters in the older population that showed a significant difference (*p* < 0.05) after food intake were VFT, SDP, DBP, HR, LA volume, E/A ratio, VTI E, VTI A, ESV, and LVEF (Table 3). There was a significant difference (*p* < 0.05) between the groups in EDV, VTI E (fasting), VTI A, and LVEF but no significant difference between the groups in MAD and VTI E (after food intake) (Table 4).

## 4. Discussion

This study showed that VFT was higher in the younger population than in the older population as expected due to the slowed relaxation of the LV due to normal aging [11]. In the older population, food intake significantly increased VFT, whereas no change was observed in the younger population. As a consequence, there were no difference in VFT after food intake between the study groups. It is difficult to standardize food intake in relation to clinical examinations. In different studies, especially with small sample sizes, it could be a factor to take into consideration.

Previous studies have shown that VFT in normal LV function is within the range of 1.8–5.5 [15,16,23]. Different pathologies, like mitral valve stenosis or other pathological conditions affecting the diameter of the mitral annulus, increases VFT. A reduced LVEF results in a significant reduction in VFT in a range between 1.5 and 2.5 [15]. VFT has also been shown to be reduced in patients with heart failure with preserved ejection fraction (HFpEF) and heart failure with reduced ejection fraction (HFrEF), compared to a healthy control group [14]. According to the formula for calculation of VFT, a decrease in β results in a higher value of VFT. Thus, a lower VTI of A-wave and a larger VTI of E-wave are advantageous, as it indicates a higher VFT value, hence a better diastolic LV function [15]. The results from this study reflect this with a higher VTI E in the younger group vs. the older group and a lower VTI A in the younger group vs. the older group. VTI E was lower and VTI A was higher before food intake in the older population just as expected due to the stiffness and impaired relaxation of the LV that follows normal aging with a prolonged late diastolic filling (higher A-wave). This might allow for the better optimization of early diastolic filling when the stroke volume increases in the older population after food intake, with a significant rise in VTI E, i.e., 17.1%, compared to the younger population, i.e., 2.5%. The same reasoning can be applied to the younger population with the late diastolic filling, VTI A, which increases significantly (16.7%) compared to the older population (5.6%).

When examining the VFT results before and after food intake in each population, there was no significant difference (*p* = 0.369 and a change of 0%) in the younger group but a significant difference (*p* < 0.001 and a change of 15%) in the older group. The reason why VFT increases in the older population after food intake may partly be explained by the VTI E value that increases 17.1% and that a larger VTI E results in higher VFT.

Overall, when examining the results in Table 2 and Table 3, there are more variables with significant differences after food intake in the older population compared to the younger population. This reflects that food intake may have a greater impact on hemodynamics of the heart in the older population. No significant differences were observed between sexes in VFT before and after food intake, which aligns with the established understanding that there are no sex differences in diastolic function.

In both groups, the median VFT was lower than reported by Gharib et al. [15,23] where a normal VFT should be between 3.3 and 5.5. However, the results of VFT presented in our study were higher than those reported by Cirovic et al. [16], where VFT was 1.8–2.6. The median (IQR) fasting value for the younger group was 2.5 (2.1–3.0) and for the older group was 2.0 (1.5–2.5). Values below the suggested normal values, however, do not exclude the formation of the vortex ring [27]. All participants in this study performed a complete echocardiographic examination in which all participants had normal LVEF, as reduced LVEF can result in lower VFT [15]. A lower VFT in the participants may indicate measurement errors and not pathological changes. Studies reporting higher values may have used different or less accurate measurement methods. However, since the measurements were repeated systematically in each participant, any measurement errors have no impact when comparing VFT before and after food intake or between the age groups in the present study.

According to previous studies, there are also different opinions about the use and importance of VFT. A previous study has shown that VFT is a potentially valuable index for distinguishing between different degrees of diastolic dysfunction and can thus provide clinically useful information about the prognosis of patients with diastolic dysfunction [17]. Other studies have suggested that VFT is not a reliable index of diastolic function due to dynamic conditions within the LV [28,29]. Stewart et al. showed that the formation of the vortex ring was affected by the intraventricular pressure difference during the early filling phase, and that VFT is not affected by changes in diastolic function [29]. Although VFT is not a measure of diastolic dysfunction, VFT can still provide useful insights into flow conditions in the LV and, as in the present study, demonstrate possible differences during different conditions.

Earlier studies have shown that food intake affects hemodynamics and different echocardiography variables [3,4,5,30,31]. The question, however, is whether these effects are clinically relevant. In our study, LA volume, E/A ratio, ESV, and LVEF showed statistically significant differences before and after food intake. All of these values were still within normal range [11,26] and would not have changed the results of the TTE examination even if there was a statistically significant difference between them before and after food intake. All study subjects were free of cardiovascular diseases, and perhaps the results would be different if participants had heart pathologies or if the meal had a different composition. The change in LA volume was modest, although statistically significant. The LA is an important marker of diastolic function and is also important from a prognostic point of view [32]. Also, LA is known to serve as a mirror for the diastolic function of the LV. It could be interesting to study the mechanical aspects in the LA due to food intake in future studies. Many consider invasive measurements the gold standard for hemodynamic assessments, including diastolic function and filling pressures [33]; however, how our findings compare to invasive data is not yet known. It should be noted that an invasive procedure has potential risks, and in clinical practice, we strive to avoid unnecessary invasive testing. We do not routinely perform such measurements solely to confirm suspected diastolic dysfunction. Instead, TTE serves as our main non-invasive tool for assessing diastolic function in the clinical context, balancing diagnostic value with patient safety and practicality.

To the best of our knowledge, there is no consensus on how to measure VFT. The method used in the present study was performed according to methodological descriptions from previous studies [14,15,16]. Some studies have calculated VFT differently [34,35,36,37], especially when calculating β where the maximum velocities of E-wave and A-wave have been used instead of VTI of E and A. Measurements of maximum velocity instead of VTI may result in different outcomes. This could be a major factor for different findings in absolute values. The aim of this investigation was, however, to assess if VFT was affected by food intake.

In the present study, MAD was calculated in early diastole in the apical four-chamber projection according to the studies conducted by Poh et al. [14] and Circovic et al. [16], instead of calculating an average diameter over the entire diastole as performed in the study conducted by Gharib et al. [15]. It was not specified when in early diastole MAD should be measured, which could affect results. In this study, MAD was measured in the early phase of diastole when the mitral valve leaflets were completely open. A study conducted by Ricci et al. [38] highlights that MAD varies during the cardiac cycle. Calculating MAD at the beginning of early diastole, instead of calculating an average diameter or in another phase of the heart cycle, may have contributed to a higher diameter and thus also lower VFT values in this study. Since the variable α is inversely proportional to VFT, this means that any measurement errors when measuring MAD also impact VFT. MAD can also be calculated by taking the average of the largest diameter obtained from the four-chamber projection, the three-chamber projection, and the two-chamber projection [15]. The method used by this study to calculate VFT was easier to perform and has been used in several previous studies [14,34], which means that the variability has been as low as possible. To avoid measurement error, which may be a major source of error in TTE examinations with subjective assessments, all measurements in this study were performed on three different heartbeats. The decision to make measurements 30 min after food intake was based on previous hemodynamic studies of the effect of food intake. There is, however, no information if this is the optimal time point. This could be an area of interest for investigations in future studies.

All assessments were tested for normality with q-plot and histograms, and since normal distribution was not reached, non-parametric statistical methods were used. For all comparisons in which Wilcoxon matched-pairs test was used, a paired samples t-test gave the same result.

## 5. Limitations

In both populations, the echocardiographic examiner was not blinded to the subjects’ food intake as a single observer performed all the examinations. Also, a control group not receiving the standardized rice pudding meal after overnight fasting was not included in the present study. When comparing the results between the younger and the older population, it is worth noting that there were two different examiners, an interval of ten years between the investigations, and different echocardiographic devices from different technological eras used. This might have affected comparison between the populations, even though the meal composition was similar. There is also a possibility for intra- and inter-vendor differences. The measurements of VFT are based on primarily two parts: the pulsed Doppler measurement of the inflow in the left ventricle and the 2D measurements of the mitral annulus. It is not likely that two different machines, following generations, from the same manufacture would differ significantly between generations. We are, however, not aware of any investigation that has evaluated these aspects. This study had a somewhat small sample size for the different groups, and we had no data on different metabolic aspects, such as insulin response profiles or blood glucose levels. Moreover, we did not perform the direct measurement of preload or splanchnic blood flow, which are the presumed mediators of the observed changes, and could have given a deeper understanding of the observed hemodynamic changes.

## 6. Conclusions

This study demonstrates a difference in VFT between younger and older individuals before food intake, but not 30 min afterwards. While VFT remained unchanged in the younger population before and after eating, a significant difference was observed in the older population. The present findings may be difficult to implement in daily clinical practice, but they suggest that the influence of food intake may be considered in studies, especially with small sample sizes. These findings also suggest that the physiological response to food intake may differ with age, which could have implications for future research. However, the clinical relevance of these results remains limited. Furthermore, the results indicate that VFT serves as an LV filling-independent index in young healthy individuals and a partially LV filling-independent index in older healthy individuals. However, no conclusions can be drawn regarding its behavior in patients with different diseases or those on medication. Further studies are warranted with data from different echocardiographic devices from different vendors. A control group not eating a standardized meal would also be valuable. Future studies on the significance of VFT as a measure of diastolic LV function are also needed.

## Figures and Tables

**Table 1:** Descriptive statistics for two healthy populations (younger and older).

	Median (IQR)	
Number of participants	23 (12 females, 11 males)	30 (15 females, 15 males)
Age (years)	25 (24–28)	68 (66–69)
Height (cm)	177 (168–184)	172 (163–181)
Weight (kg)	65 (58–80)	75 (59–82)
BSA (m^2^)	1.8 (1.7–2.0)	1.9 (1.6–2.0)
BMI (kg/m^2^)	21.6 (20.2–23.1)	24.3 (22.1–26.0)

BSA—body surface area; BMI—body mass index; IQR—interquartile range.

**Table 2:** Statistics for the younger population (*n* = 23). The different variables measured and their median value and interquartile range (IQR) for both fasting and 30 min after food intake, and *p*-value < 0.05 from the Wilcoxon matched-pairs test was considered statistically significant. Percent change for the different values for fasting versus 30 min after food intake are presented.

Variable	Fasting Median (IQR)	30 min After Food Intake Median (IQR)	*p*-Value	Percent Change Fasting Versus 30 min (%)
SBP (mm Hg)	103 (97–110)	103 (93–110)	0.760	0
DBP (mm Hg)	65 (62–70)	58 (52–62)	<0.001	−11
HR (bpm)	60 (51–68)	64 (55–72)	0.009	7
LA volume (mL)	48.0 (43.5–58.1)	52.3 (47.8–61.0)	0.010	8.9
E/A ratio	1.8 (1.6–2.2)	1.7 (1.5–2.0)	0.104	−5.6
E/é ratio (average)	5.1 (4.5–5.7)	5.2 (4.7–5.8)	0.456	2.0
VTI E (cm)	11.8 (10.8–13.5)	12.1 (11.2–13.3)	0.761	2.5
VTI A (cm)	4.2 (3.4–5.0)	4.9 (4.2–6.1)	0.002	16.7
MAD (cm)	3.0 (2.8–3.2)	3.0 (2.8–3.2)	0.878	0
EDV (mL)	130.7 (104.3–151.4)	131.0 (112.8–153.4)	0.101	0.2
ESV (mL)	55.7 (49.0–70.7)	54.0 (49.0–62.3)	0.153	−3.1
LVEF (%)	54 (52.5–57.3)	56.4 (54.2–59.9)	<0.001	4.4
VFT	2.5 (2.1–3.0)	2.5 (2.2–3.1)	0.369	0

SBP—systolic blood pressure; DBP—diastolic blood pressure; HR—heart rate; LA—left atrium; VTI—velocity time integral; E—peak of early diastolic mitral flow velocities; A—late diastolic mitral flow velocities; e’—pulsed tissue Doppler velocities; MAD—mitral annulus diameter; EDV—end-diastolic volume; ESV—end-systolic volume; LVEF—left ventricular ejection fraction; VFT—vortex formation time.

**Table 3:** Statistics for the older population (*n* = 30). The different variables measured and their median value and interquartile range (IQR) for both fasting and 30 min after food intake, and *p*-value < 0.05 from the Wilcoxon matched-pairs test was considered statistically significant. Percent change for the different values for fasting versus 30 min after food intake are presented.

Variable	Fasting Median (IQR)	30 min After Food Intake Median (IQR)	*p*-Value	Percent Change Fasting Versus 30 min (%)
SBP (mm Hg)	127 (116–137)	124 (112–130)	0.021	−2
DBP (mm Hg)	78 (74–84)	72 (66–75)	<0.001	−8
HR (bpm)	61 (57–67)	65 (60–68)	0.009	7
LA volume (mL)	53.7 (44.6–65.8)	54.2 (49.4–67.2)	0.012	1
E/A ratio	1.0 (0.8–1.1)	1.0 (0.8–1.2)	0.039	0
E/é ratio (average)	7.6 (6.5–8.9)	7.8 (6.7–8.6)	0.371	2.6
VTI E (cm)	10.5 (9.0–11.7)	12.3 (10.6–14.8)	<0.001	17.1
VTI A (cm)	7.1 (6.2–7.5)	7.5 (6.8–8.3)	<0.001	5.6
MAD (cm)	2.9 (2.7–3.1)	2.9 (2.7–3.1)	0.935	0
EDV (mL)	90.4 (81.6–111.5)	91.1 (77.3–113.6)	0.453	0.8
ESV (mL)	32.5 (27.7–41.0)	29.6 (22.1–36.4)	<0.001	−9.0
LVEF (%)	64.9 (62.3–66.3)	68.9 (66.6–72.0)	<0.001	6.2
VFT	2.0 (1.5–2.5)	2.3 (1.5–2.9)	<0.001	15

SBP—systolic blood pressure; DBP—diastolic blood pressure; HR—heart rate; LA—left atrium; VTI—velocity time integral; E—peak of early diastolic mitral flow velocities; A—late diastolic mitral flow velocities; e’—pulsed tissue Doppler velocities; MAD—mitral annulus diameter; EDV—end-diastolic volume; ESV—end-systolic volume; LVEF—left ventricular ejection fraction; VFT—vortex formation time.

**Table 4:** Medians, interquartile ranges (IQRs), and *p*-values (<0.05 was considered statistically significant) from the Mann–Whitney U test for the comparison between the older and the younger population regarding vortex formation time (VFT) and the other parameters involved in the equation used when calculating VFT.

Variable	Fasting			30 min After Food Intake		
	Younger Population Median (IQR)	Older Population Median (IQR)	*p*-Value	Younger Population Median (IQR)	Older Population Median (IQR)	*p*-Value
MAD (cm)	3.0 (2.8–3.2)	2.9 (2.7–3.1)	0.193	3.0 (2.8–3.2)	2.9 (2.7–3.1)	0.122
VTI E (cm)	11.8 (10.8–13.5)	10.5 (9.0–11.7)	0.005	12.1 (11.2–13.3)	12.3 (10.6–14.8)	0.851
VTI A(cm)	4.2 (3.4–5.0)	7.1 (6.2–7.5)	<0.001	4.9 (4.2–6.1)	7.5 (6.8–8.3)	<0.001
EDV (mL)	130.7 (104.3–151.4)	90.4 (81.6–111.5)	<0.001	131.0 (112.8–153.4)	91.1 (77.3–113.6)	<0.001
LVEF (%)	54 (52.5–57.3)	64.9 (62.3–66.3)	<0.001	56.4 (54.2–59.9)	68.9 (66.6–72.0)	<0.001
VFT	2.5 (2.1–3.0)	2.0 (1.5–2.5)	0.011	2.5 (2.2–3.1)	2.3 (1.5–2.9)	0.172

MAD—mitral annulus diameter; VTI—velocity time integral; E—peak of early diastolic mitral flow velocities; A—late diastolic mitral flow velocities; EDV—end-diastolic volume; LVEF—left ventricular ejection fraction; VFT—vortex formation time.

## Data Availability

Please contact author for data requests.
